# Effect of fluoride gels with nano-sized sodium trimetaphosphate on the *in vitro* remineralization of caries lesions *

**DOI:** 10.1590/1678-7757-2023-0115

**Published:** 2023-06-26

**Authors:** Mariana Emi NAGATA, Alberto Carlos Botazzo DELBEM, Liliana Carolina BÁEZ-QUINTERO, Marcelle DANELON, Caio SAMPAIO, Douglas Roberto MONTEIRO, Annette WIEGAND, Juliano Pelim PESSAN

**Affiliations:** 1 Universidade Estadual Paulista Faculdade de Odontologia Departamento de Odontologia Preventiva e Restauradora Araçatuba São Paulo Brasil Universidade Estadual Paulista (UNESP), Faculdade de Odontologia, Departamento de Odontologia Preventiva e Restauradora, Araçatuba, São Paulo, Brasil.; 2 Universidade do Oeste Paulista Programa de Pós-graduação em Ciências da Saúde Presidente Prudente São Paulo Brasil Universidade do Oeste Paulista (UNOESTE), Programa de Pós-graduação em Ciências da Saúde, Presidente Prudente, São Paulo, Brasil.; 3 University Medical Center Göttingen Department of Preventive Dentistry, Periodontology and Cariology Göttingen Germany University Medical Center Göttingen, Department of Preventive Dentistry, Periodontology and Cariology, Göttingen, Germany.

**Keywords:** Fluorides, Phosphates, Dental Enamel, Nanoparticles, Nanotechnology

## Abstract

**Objective:**

To evaluate the effects of fluoride (F) gels supplemented with micrometric or nano-sized sodium trimetaphosphate (TMPmicro and TMPnano, respectively) on the *in vitro* remineralization of caries-like lesions.

**Methodology:**

Bovine enamel subsurface lesions (n=168) were selected according to their surface hardness (SH) and randomly divided into seven groups (n=24/group): Placebo (without F/TMP), 4,500 ppm F (4500F), 4500F + 2.5% TMPnano (2.5% Nano), 4500F + 5% TMPnano (5% Nano), 4500F + 5% TMPmicro (5% Micro), 9,000 ppm F (9000F), and 12,300 ppm F (Acid gel). The gels were applied in a thin layer for one minute. Half of the blocks were subjected to pH cycling for six days, whereas the remaining specimens were used for loosely- (calcium fluoride; CaF_2_) and firmly-bound (fluorapatite; FA) fluoride analysis. The percentage of surface hardness recovery (%SHR), area of subsurface lesion (ΔKHN), CaF_2_, FA, calcium (Ca), and phosphorus (P) on/in enamel were determined. Data (log_10_-transformed) were subjected to ANOVA and the Student-Newman-Keuls’ test (p<0.05).

**Results:**

We observed a dose-response relation between F concentrations in the gels without TMP for %SHR and ΔKHN. The 2.5% Nano and 5% Micro reached similar %SHR when compared with 9000F and Acid gels. For ΔKHN, Placebo and 5% Nano gels had the highest values, and 5% Micro, 2.5% Nano, 9000F, and Acid gels, the lowest. All groups had similar retained CaF_2_ values, except for Placebo and Acid gel. We verified observed an increase in Ca concentrations in nano-sized TMP groups. Regarding P, TMP groups showed similar formation and retention to 9000F and Acid.

**Conclusion:**

Adding 2.5% nano-sized or 5% micrometric TMP to low-fluoride gels lead to enhanced *in vitro* remineralization of artificial caries lesions.

## Introduction

Caries prevention programs focus on children and usually considered as a priority for dental public health since they are less expensive than treatment.^1^ Topically applied fluoride (F) products, especially at high concentrations, have served as a low-cost and easily implemented measure to prevent and treat caries lesions,^2^ most commonly in solutions, gels, and varnishes.^3^ Because of the risk of overingestion and thus acute toxicity, recommendations usually suggest the use of F gels for children older than six years old.^4^ Considering the extensive benefits and wide use of these products, the search for strategies that increase their clinical effectiveness without increasing possible side-effects in young children is highly desirable.

We find considerable *in vitro* and *in situ* evidence on the efficacy of fluoridated products supplemented with phosphate salts on enamel de- and remineralization processes, including sodium trimetaphosphate (TMP),^5 , 6^ sodium hexametaphosphate (HMP),^7 , 8^ and calcium glycerophosphate (CaGP).^9 , 10^ A randomized clinical trial has recently confirmed laboratory evidence, showing that the supplementation of a low-F (500 ppm F) toothpaste with micrometric TMP or CaGP resulted in lower or similar caries progression, respectively, than a conventional dentifrice formulation (1100F).^11^

In addition, the use of nano-sized TMP or HMP further enhanced the effects of F on enamel de- and remineralization when added to dentifrices.^12 , 13^ Such additional effects could be due to the high ratio of surface area to volume, as well as a higher percentage of atoms on surfaces than on larger particles, which makes nano-sized particles more reactive.^13^

Despite the promising results obtained for dentifrice formulations described above, no evidence is available for F vehicles for professional application supplemented with nano-sized phosphate salts. Considering the relevance of this issue for clinical practice, this study aimed to evaluate the effects of F gels supplemented with two sizes of sodium trimetaphosphate (TMP) particles (conventional: TMPmicro or TMP nanoparticles: TMPnano) on the *in vitro* remineralization of caries-like enamel lesions. Our null hypotheses suggested that (1) fluoride gels supplemented with TMP would promote similar remineralization to gels containing the same F concentrations without this phosphate and that (2) varnishes containing TMPmicro or TMPnano would promote similar remineralization.

## Methodology

### Experimental Design

**Figure 1:**
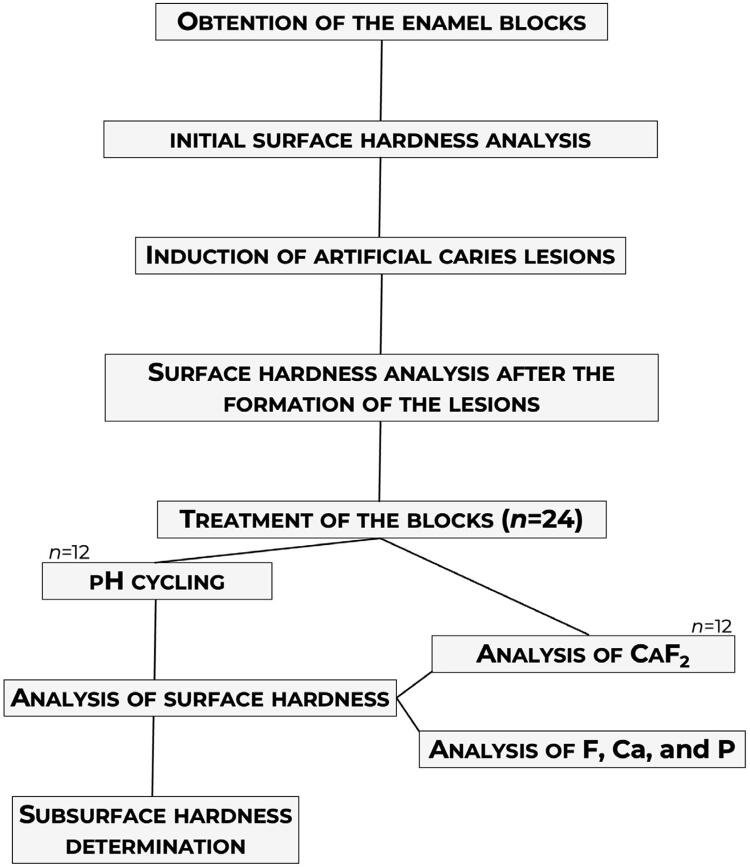
Schematic diagram summarizing the experimental design of this study

Bovine enamel blocks (4×4 mm, *n* =168) were obtained from the flattest portion of the vestibular face of crowns. The blocks were selected by surface hardness (SH) and caries-like lesions were induced. The lesioned blocks were randomly divided into seven experimental groups ( *n* =24/group), one for each tested gel: (a) Placebo (without F or TMP), (b) 4,500 ppm F (4500F), (c) 9,000 ppm F (9000F), (d) 4500F plus 2.5% nano-sized TMP (2.5% Nano), (e) 4500F plus 5% nano-sized TMP (5% Nano), (f) 4500F plus 5% micrometric TMP (5% Micro), and (g) 12,300 ppm F (acid gel). The blocks were treated once with the respective gels before a pH cycling regimen (six days). SH; integrated area of the subsurface lesion (ΔKHN); enamel F, calcium (Ca), phosphorus (P) concentrations; and calcium fluoride (CaF_2_) formed (after gel application) and retained (after pH cycling) in/on enamel were determined. Figure 1 summarizes the experimental design of this study.

### Synthesis and characterization of nano-sized (TMP) particles

Nano-sized TMP were synthesized and characterized at the Federal University of São Carlos, as previously described.^13^ The particles were prepared by ball milling 70 g of pure (micrometric) sodium trimetaphosphate (Na_3_O_9_P_3_, Aldrich, purity ≥ 95% CAS 7785- 84-4) using 500 g of zirconia spheres (diameter 2 mm) in 1 L of isopropanol. After 48 h, the resulting powder was separated from the alcoholic medium and ground in a mortar. Powder crystallinity was characterized by X-ray diffraction (XRD) using a Rigaku Dmax 2500 PC diffractometer in the 2θ range from 10° to 80° at a scanning rate of 28/min. The coherent crystalline domains (crystallite size) were estimated using the Scherrer equation: $L=\lambda K/B\cos\theta_{B}$ , in which *L* is the linear dimension of a monocrystalline nano-particle, ƛ is the wavelength of the incident X-ray, *B* is the diffraction line width of the diffraction peak, θ_B_ is the Bragg angle obtained from the XRD pattern, and the shape factor *K* is a numerical constant whose value is 0.9. Scanning electron microscopy (SEM) images were collected using a Philips XL-30 FEG. A micrometric TMP particle size of 450 nm was observed, reaching an approximate size of 22.7 nm (nanoparticle).

### Gel formulation and determination of fluoride in products

The experimental gels were produced in the Pediatric Dentistry laboratory at the Araçatuba Dental School, using the following ingredients: carboxymethylcellulose (Sigma-Aldrich Co., St. Louis, MO, USA), sodium saccharin (Vetec, Duque de Caxias, Rio de Janeiro, Brazil), glycerol (Merck, Darmstadt, Germany), peppermint oil (Synth), and water. A fluoride-free placebo and two treatment gels with F (NaF - Merck^®^, Germany) at the concentrations of 4,500 and 9,000 ppm F were prepared. Micrometric TMP (Sigma-Aldrich Co., St. Louis, MO, USA) was added at a concentration of 5% and nano-sized TMP at concentrations of 2.5% and 5% to the 4,500 ppm F gel. A commercial acidic gel was used as a positive control (12,300 ppm F, acid gel, pH = 4.5, DFL Indústria e Comércio S.A., Rio de Janeiro, RJ, Brazil). F concentrations of the gels was determined using a specific electrode for the F^−^ ion (9609 BN; Orion Research Inc., Beverly, MA, USA) attached to an ion analyzer (Orion 720 A+; Orion Research Inc.) and calibrated with standards containing 0.125–2.0 ppm F. Approximately 100–110 mg of each product was dissolved in deionized water and transferred to a volumetric flask. The volume was then adjusted to 100 ml using deionized water. For each product, three dilutions were prepared, which were analyzed in duplicate, after buffering with total ionic strength adjustment buffer II (TISAB II).^14^

### Induction of artificial caries lesions

To induce caries-like lesions, all surfaces of each specimen (except the enamel surface) were coated with acid-resistant varnish and subsurface enamel demineralization was produced by immersing each block individually in 32 mL of a solution containing 1.3 mmol/L Ca and 0.78 mmol/L P in 0.05 mol/L acetate buffer, pH 5.0, 0.03 ppm F, for 16 h at 37 °C.^15 , 16^ The blocks were subjected to post-demineralization surface hardness measurement (SH_1_) by producing five indentations spaced 100-μm apart from the five initial ones (SH).

### Treatment with gels and pH cycling (Re>Des)

In the first day of pH cycling, the exposed enamel area was completely covered by a thin layer of gel, applied using a cotton swab for 1 min. After treatment, the gel was removed and blocks were washed with deionized water and gently dried with absorbent paper. The specimens were individually subjected to a pH cycling regimen at 37 °C for six consecutive days. Each cycle alternated between the remineralizing (RE) solution (1.1 mL/mm^2^; 1.5 mmol/L Ca, 0.9 mmol/L P, 150 mmol/ L KCl in cacodylate buffer 20 mmol/L, 0.05 ppm F, pH 7.0) and the demineralizing (DE) solution for cariogenic challenges (2.2 mL/mm^2^; 2.0 mmol/L Ca and P, in acetate buffer 75 mmol/L, 0.04 ppm F, pH 4.7).^17^ In brief, each cycle comprised a sequence of immersion in RE (4 h), DE (2 h), RE (4 h), and a freshly prepared RE (16 h).

### Hardness analysis

Surface hardness (SH) was measured before the experiments, after enamel demineralization (SH_1_), and after pH cycling (SH_2_) using a hardness tester (Buehler, Lake Bluff, USA and Mitutoyo Corporation, Kanagawa, Japan) and a Knoop diamond indenter under a 25-g load for 10 s.^13^ In total, five indentations, spaced 100 μm apart, were made near the center of the enamel surface to determine initial surface hardness (SH) and five more indentations were made after artificial caries lesions were induced (to measure SH_1_), 100 μm apart from the SH indentations. After the experimental periods, five further indentations were made to measure SH_2_, spaced 100 μm apart from the SH_1_ indentations. The percentage of surface hardness recovery (%SHR) was estimated using the following formula: %SHR=[(SH2−SH1)/(SH−SH1)]×100 . To measure the area of the subsurface lesions, enamel blocks were longitudinally sectioned through their center and embedded in acrylic resin with their cut face exposed and polished. A sequence of 14 indentations at 5, 10, 15, 20, 25, 30, 40, 50, 70, 90, 110, 130, 220, and 330 μm from the enamel surface were created in the central region, using the aforementioned microhardness tester with a Knoop diamond indenter under a 5-g load for 10 s (Buehler, Lake Bluff, USA).^18^ The integrated hardness area (KHN×μm) for the lesion into sound enamel was estimated using the trapezoidal rule (GraphPad Prism, version 3.02) and subtracted from the integrated hardness area of healthy enamel to obtain the integrated recovery of subsurface hardness (ΔKHN).^18^

### Determination of CaF2-like concentrations (formed and retained)

The concentration of (CaF_2_) on enamel was determined immediately after the application of the experimental gels (to determine the formed CaF_2_) and after pH cycling (to evaluate the retained CaF_2_).^19^ The blocks were measured with a Mitutoyo CD-15B digital caliper (Mitutoyo Corp., Japan) to obtain the surface areas of the specimens. The surface of each specimen (except for the enamel) was coated with wax and subsequently immersed in 0.5 ml of a 1.0 mol/l KOH solution for 24 h under constant agitation. Then, the solution was neutralized with 0.5 ml of 1.0 mol/l HCl and buffered with 1.0 ml of TISAB II. An ion analyzer (720A; Orion Research, USA) and a combined ion-selective electrode (9609 BN; Orion Research, USA) previously calibrated with standards at 0.0625, 0.125, 0.250, 0.500, and 1.0 ppm F were used to measure CaF_2_ concentration. Measurements were obtained in mV and converted to microgram F per square centimeter in Microsoft Excel.^19^

### Fluoride, calcium, and phosphorus content in enamel (formed and retained)

The other halves of the blocks were sectioned again (2×2 mm) and enamel biopsies were performed.^20 , 21^ The blocks were fixed to a mandrel and attached to the top of a modified microscope with a micrometer (Pantec, São Paulo, Brazil) to measure their depth. Self-adhesive polishing disks (13 mm in diameter) with 400-grit silicon carbide (Buehler) were fixed to the bottom of polystyrene crystal tubes (J-10, Injeplast, São Paulo, Brazil). A 50 μm-deep layer was removed from each enamel block^9 , 18^ and 0.8 mL HCl 0.5 mol/L was added to the resulting enamel powder. The tubes were agitated for 60 min and 0.8 mL NaOH 0.5 mol/L was then added to them, following a modified protocol based on Danelon, et al.^13^ (2015). For F analysis, samples were buffered with TISAB II and analyzed with an ion-specific electrode (Orion 9609) connected to an ion analyzer (Orion 720+). A 1:1 ratio (TISAB:sample) was used. Ca analysis was performed using the Arsenazo III colorimetric method.^22^ P was measured using the method described by Fiske and Subbarow.^23^

### Statistical analysis

All data passed normality (Shapiro-Wilk) and homogeneity (Barlett) tests after log_10_-transformation, obeying a normal and homogeneous distribution. Data on SH_1_, %SHR, and ΔKHN were subjected to one-way ANOVA followed by the Student–Newman–Keuls *post hoc* test, considering our study groups (seven levels) as variation factors. For enamel CaF_2_, F, Ca, and P contents, data were subjected to two-way ANOVA, considering our study groups (seven levels) before (formed) and after (retained) pH cycling (two levels) as variation factors, followed by the Student–Newman–Keuls *post hoc* test. All statistical analyses were performed using the SigmaPlot 12.0 software (Systat Software Inc., San Jose, CA, USA), adopting a *p* <0.05.

## Results

Mean fluoride concentrations (Standard Deviation) in the placebo, 4500F, 9000F, 2.5% Nano, 5% Nano, Micro 5%, and Acid gel groups averaged 19.8 (2.91), 4339.9 (96.7), 9120.7 (114.4), 4383.2 (180.3), 4093.9 (224,6), 4148,7 (93.2), and 13136.9 (995.07) ppm F, respectively. Mean (SD) initial surface hardness was 366.3 KHN (5.1), whereas mean (SD) surface hardness after demineralization (SH1) was 58.9 KHN (12.6), with no statistically significant difference among the groups after random allocation ( *p* =0.080). All gels, except for the acidic one, had a neutral pH.

We observed a dose-response relation between fluoride concentrations in the experimental gels without TMP and %SHR (Placebo < 4500F < 9000F = acid gel). The lowest %SHR values were observed for placebo, 4500F, and 5% Nano, which significantly differed from the other groups. Conversely, the 5% Micro, 2.5% Nano, 9000F, and Acid gel groups had the highest %SHR values but no significant differences between them. We found a pattern in subsurface lesion areas (ΔKHN) that resembled that we observed for %SHR, with the placebo and 5% Nano gels ( *p* =0.953) showing significantly higher ΔKHN values than the remaining groups, without significant differences among the 5% Micro, 2.5% Nano, 9000F, and acid gel groups. Adding 5% TMPnano to 4500F led to results that resembled those for the placebo gel ( *p* =0.953; Table 1 ).

**Table 1:** Mean (SD) percentage of surface hardness recovery (%SHR) and integrated loss of subsurface hardness (ΔKNH) according to groups

Groups	%SHR	ΔKHN
Placebo	14.9 (3.5)^a^	5795.9 (1176.7)^a^
4,500	24.2 (6.6)^b^	4873.2 (955.3)^b^
9,000	29.8 (8.4)^c^	3661.5 (646.1)^c^
4,500 5% TMP micro	34.8 (6.7)^c^	3063.8 (959.8)^c^
4,500 2.5% TMP nano	35.3 (9.1)^c^	3136.4 (884.2)^c^
4,500 5% TMP nano	19.7 (5.8)^d^	5773.2 (1070.2)^a^
Acid gel	36.9 (6.0)^c^	3132.5 (789.2)^c^

Distinct superscript lowercase letters indicate statistical significance in each column (Student-Newman-Keuls’ test, n=12, p<0.05). Data were log_10_-transformed for statistical analyses.

**Figure 2:**
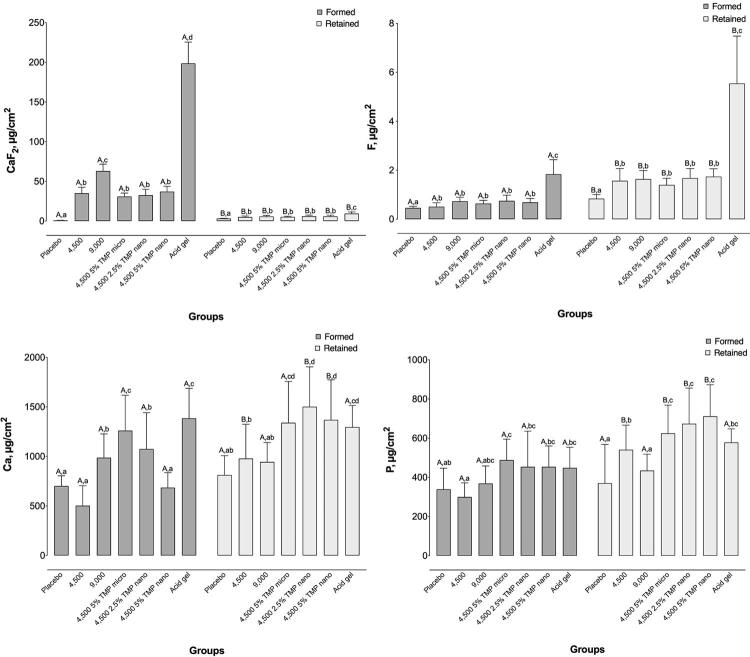
Mean calcium fluoride (CaF2), firmly bound fluoride (F), calcium (Ca), and phosphorous (P) formed and retained on/in enamel after gel treatment and pH-cycling according to groups. Bars denote mean standard deviations. Different lowercase superscript letters show significant differences betweeen groups in each analysis. Different uppercase superscript letters indicate differences between CaF2, F, Ca and P – formed and retained) within each group. Two-way ANOVA (log10-transformed data) and Student-Newman-Keuls’ test, n=12 (p<0.05)

Acid gel promoted the highest CaF_2_-like levels out of all groups after topical application ( *p* <0.001). After pH cycling, all groups had similar CaF_2_-like concentrations, except for placebo and acid gel ( *p* <0.001). The 9,000 and TMP groups showed similar concentrations of formed F in enamel. The amount of F retained in enamel significantly increased after remineralization in all groups ( *p* <0.05) ( Figure 2 ).

As for enamel Ca concentrations, we observed a marked increase after pH cycling for groups treated with nano-sized TMP, in contrast to the other groups ( *p* <0.05). Regarding formed and retained P in enamel, groups treated with TMP showed similar values to 9000F and acid gel. P levels increased after pH cycling, despite being significant only for the 4500F, 5% Micro, 2.5% Nano, and 5% Nano treatments ( *p* <0.05). Figure 2 shows other comparisons.

## Discussion

Proposals to add inorganic phosphate salts to topical F products aim to improve the efficacy of the prevention and treatment of early caries lesions (i.e., white spot lesions) and reduce F levels in formulations to minimize possible acute side-effects. *In vitro* and *in situ* studies showed that micrometric TMP associated with fluoridated gels significantly enhanced the remineralization of artificial caries lesions^24^ and prevented enamel demineralization.^25 , 26^ This study showed that adding TMP to a low-F gel (4500F) resulted in a higher remineralizing capacity then its counterpart without TMP, achieving levels similar to those of conventional neutral (9,000 ppm F) and acid (12,300 ppm F) gels. Nonetheless, we achieved no additional benefit by using nano-sized TMP, when we compared it with micrometric particles, thus leading us to accept our second null hypothesis.

The current method and the product tested were chosen based on promising results observed for dentifrices associated with nano-sized TMP and HMP particles. Danelon, et al.^27^ (2017) found that *in vitro* treatment with a 1100 ppm F toothpaste supplemented with 3% TMPnano reduced mineral loss in ~44%, compared to its micrometric counterpart. In an *in vitro* study, Dalpasquale, et al.^12^ (2017) showed that adding 0.5% HMPnano to a 1100F toothpaste significantly enhanced its effects against enamel demineralization compared to its counterpart without HMPnano. Furthermore, adding 0.05% nano-sized TMP to a low-F toothpaste (250 µg F/g) promoted significantly lower ΔKHN among all groups, including a 1100 ppm F toothpaste.^28^

In this study, while 2.5% TMPnano, 5% TMPmicro, 9,000F, and acid gel promoted similar %SHR and ΔKHN, the addition of 5% TMPnano to 4500F resulted in %SHR and ΔKHN values similar to those achieved for the placebo formulation. These findings seem to confirm that TMP:F molar ratios have a strong influence on the resulting effect against enamel demineralization.^27 , 29 - 31^ The TMPnano concentrations tested in this study (2.5% and 5%) were based on TMP:F ratio from previous studies which reported that adding micrometric particles of TMP at 5% to a 4,500 ppm F gel promoted a significantly higher effect against enamel demineralization and on the remineralization of caries-like lesions than their counterparts without TMP.^5 , 23 , 25^ Furthermore, the studies with nano-sized TMP/HMP in dentifrices were decisive for our choice of 2.5% TMPnano since we expected that a lower concentration of nano-sized TMP (compared with micrometric particles) could result in greater efficacy, as observed in previous studies.^12 , 28^

Despite most clinical studies adopting professional topical gel application times ranging from 2 to 10 min,^4^ this study used a time of 1 min based on Delbem, et al.^18^ (2010) and Villena, Tenuta, and Cury^32^ (2009). In the latter study, results showed that applying acidulated phosphate fluoride for either one or four minutes equally increased enamel F concentrations and reduced enamel demineralization. Another recommendation associated with gel application is to refrain from eating and drinking for at least 30 min after application.^4^ In this study, however, the blocks were washed with deionized water immediately after application, based on previous findings showing that water rinsing after professionally applied topical fluoride gel or foam will have no adverse effect on the therapeutic benefit of a treatment.^18 , 33^ Moreover, we adopted this step to avoid contaminating cycling solutions with gel treatments and to ensure that no trace of gel remained on the block surface, which could alter study results.

The amount of CaF_2_ formed after applying high fluoride concentrations is paramount for achieving maximum preventive and therapeutic effects since this layer acts as an efficient source of free fluoride and calcium ions during cariogenic challenges. The acid gel group had the highest formed CaF_2_-like concentrations (after topical application), followed by the 9000F and 4500F groups, confirming that CaF_2_ formation could increase by raising the fluoride concentration of the topical agent and/or lowering the pH of the topical agent.^34^ After pH cycling, all groups had similar concentrations of retained CaF_2_-like products, except the placebo and acid gel groups. Results suggest that the effect of TMP was unrelated to the deposition of calcium fluoride (CaF_2_) the enamel; this reduction might be due to an increase in Ca and F retention on the TMP molecules that are adsorbed to enamel,^6^ instead of the deposition of CaF_2_ globules on the surface of the enamel.

Nevertheless, for firmly bound F (formed), we observed similar values between the 9000F and TMP groups, and, after pH cycling, F concentrations significantly increased in all groups ( *p* <0.05), disagreeing with the findings in Manarelli, et al.^6^ (2014), a study with fluoridated varnishes. Also, we observed a marked increase in Ca concentrations in enamel after pH cycling for the nano-sized TMP groups, in contrast with groups without TMP supplementation. Regarding P results, groups treated with TMPmicro/nano showed values similar to the 9000F and acid gel groups. Moreover, adding TMP to 4,500F gel had an effect on enamel mineral composition. Despite the two- to three-fold difference in F content between TMPmicro/nano groups and 9,000F/acid gel, respectively, we observed similar F (formed), Ca, and P values in the enamel treated with these gels, which confirm the results obtained for previous studies with dentifrices.^18 , 28 , 35 , 36^

In this study, while TMPnano at 5% increased the amount of F (formed), Ca, and P in enamel, it promoted the lowest %SHR between the fluoridated gels and similar ΔKHN, when compared with the placebo gel. We could suggest that a large percentage of TMPnano would supersaturate the enamel surface and, since the adsorption of polyphosphates to enamel occurs rapidly after exposure and is followed by the adsorption of F,^37^ an appropriate molar proportion between TMP and F must be sought to optimize the anticaries action. The suggested molar proportion of TMP/NaF lies from 1.24:1 to 3.72:1.^30^ For high-fluoride gels, the 4,500 5% TMPmicro (TMP: NaF/0.7) achieved the greatest effect on enamel demineralization.

Although *in vitro* conditions can be carefully controlled and show a dose-response relation to different levels of fluoride in demineralization and remineralization processes, the main role of *in vitro* methods is to provide information about the mode of action of new compounds and facilitate the generation of sufficient quantitative data to give investigators confidence to properly design clinical trials.^38^

## Conclusion

Under the conditions of this *in vitro* study, we can conclude that adding TMP to low-fluoride gels leads to enhanced remineralization of artificial caries lesions *in vitro* and that we obtained no additional benefit by using nano-sized particles instead of micrometric ones.
